# Involvement of KLRK1 in immune infiltration of head and neck squamous cell carcinoma correlates with favorable prognosis

**DOI:** 10.1097/MD.0000000000034761

**Published:** 2023-08-11

**Authors:** Haosheng Tan, Huaiyu Yang, Jiaxin Qian, Shaoyan Liu, Dangui Yan, Liyuan Wei, Wensheng Liu

**Affiliations:** a Department of Head and Neck Surgical Oncology, National Cancer Center/National Clinical Research Center for Cancer/Cancer Hospital, Chinese Academy of Medical Sciences and Peking Union Medical College, Beijing, China.

**Keywords:** HNSCC, KLRK1 immune infiltration, immune infiltration KLRK1, prognosis

## Abstract

Head and neck squamous cell carcinoma (HNSCC) is a malignancy commonly found in the head and neck region, with a low 5-year survival rate. Although immunotherapy has made significant progress, its efficacy in HNSCC treatment remains unsatisfactory. Killer cell lectin-like receptor K1 (KLRK1), a marker highly expressed in immune cells, can bind to its ligands expressed by cancer cells to exert its antitumor effect. However, the role of KLRK1 in HNSCC has yet to be studied extensively. This study aimed to explore the involvement of KLRK1 in immune infiltration of HNSCC and its correlation with prognosis. We analyzed KLRK1 expression data from the Cancer Genome Atlas database. The relationship between KLRK1 and immune cell infiltration has also been investigated. Finally, we analyzed the association between the expression of KLRK1 and its ligands and the prognosis of patients with HNSCC. We found that KLRK1 was highly expressed in HNSCC and correlated with better prognosis. KLRK1 expression was correlated with age, histological grade, HPV infection, pT, pN, pTNM stage, primary site, and survival status. High expression levels of KLRK1 have been linked to high levels of immune cell infiltration, particularly CD4/8 (+) T lymphocytes. Among the ligands of KLRK1, UL16 binding protein (ULBP) 1-3 showed high expression, which was associated with an increased risk of death. Notably, the expression of KLRK1 was negatively correlated with ULBP1-3. Patients with high levels of ULBP2/3 expression in tonsil carcinoma had poorer prognosis than those with low levels (*P* < .01), whereas ULBP1 expression levels had no significant effect on tonsil carcinoma prognosis (*P* = .770). The expression levels of ULBP1/3 were correlated with worse prognosis in patients with laryngeal cancer (*P* < .05), whereas there was no significant correlation between ULBP2 expression levels and overall survival (*P* = .269). Our study revealed that KLRK1 is highly expressed in HNSCC and is associated with a better prognosis and immune infiltration. Patients with high expression of KLRK1 ligands exhibited worse prognoses, possibly because of the expression of more soluble ligands.

## 1. Introduction

Head and neck squamous cell carcinoma (HNSCC) is one of the most prevalent malignancies of the head and neck region. In recent years, its incidence has been increasing, with 890,000 new cases reported worldwide in 2018. HNSC currently ranks as the seventh most common type of cancer globally.^[1]^ Currently, the primary treatment methods for HNSCC include surgical resection, radiotherapy (RT), and chemotherapy. Despite gradual improvements in these methods, prognosis remains poor, with a 5-year survival rate of less than 40%, and these methods are associated with a high rate of adverse events.^[2]^

Recent studies have reinforced the notion that the immune system plays a vital role in the initiation and progression of tumors and that there exists a close interdependence between tumor prognosis and the immune status of an individual.^[3]^ Immune-based interventions in the form of immunotherapy have emerged as cutting-edge approaches for tumor prognosis and treatment. Immunotherapy is a therapeutic approach that harnesses a patient’s immune system to recognize and combat tumor cells. Compared to traditional chemotherapy and radiotherapy, immunotherapy produces fewer toxic side effects. In recent years, with ongoing research on immune therapy and technological advancements, it has demonstrated notable effectiveness in the treatment of various forms of cancer.^[4]^ A recent clinical trial demonstrated that treating patients with head and neck squamous cell carcinoma using the programmed cell death protein 1 (PD-1) receptor inhibitor nivolumab resulted in significant improvements in progression-free survival and fewer adverse reactions.^[5]^

Despite considerable advancements in immunotherapy in recent years, a large proportion of patients do not respond to this treatment. This highlights the importance of identifying specific biomarkers that can accurately predict a patient’s response to immunotherapy.^[6,7]^ The killer cell lectin-like receptor (KLR) gene family encodes members of the immunoglobulin superfamily.^[8]^ This gene family has been identified in humans and many other species, and includes receptor molecules that play crucial roles in immune regulation, such as killer cell immunoglobulin-like receptor, natural killer group 2 (NKG2), and leukocyte immunoglobulin-like receptor. These receptors are widely distributed on the surface of various immune cells, including natural killer (NK) cells, T cells, B cells, and dendritic cells, thus aiding in immune surveillance and responses. The activation of killer cell lectin-like receptor K1 (KLRK1), also known as natural killer group 2D (NKG2D), within the KLR family, enables it to bind to KLRK1 ligands (NKG2DLs) present on the cancer cell surface, resulting in the activation of a variety of immune cells, including NK cells and CD8(+) T cells.^[9]^ This immune response ultimately leads to death of cancer cells. This demonstrates the potential of KLRK1/NKG2DLs as promising therapeutic targets for more effective antitumor treatment.^[10]^ However, currently there is still relatively few studies have been conducted on KLRK1 and its ligands in HNSCC. Mele et al discovered that the infiltration of NK cells in HNSCC exhibits a unique phenotype characterized by lower expression levels of KLRK1.^[11]^ A study on oral cancer revealed that the co-cultivation of NK cells and oral squamous cancer cell lines can boost the expression of KLRK1 in NK cells.^[12]^ Li et al^[13]^ have discovered, In an in vitro study using an HNSCC mouse model, Li et al^[12]^ discovered that removing the inhibition of KLRK1 can improve the effectiveness of chemotherapy for head and neck squamous cell carcinoma. However, these results showed discrepancies, indicating that the underlying mechanism of KLRK1 in HNSCC requires further investigation.

The objective of this study was to provide novel insights into the role of KLRK1 in the assessment of prognosis and immunotherapy in patients with HNSCC. This will be achieved by analyzing the expression levels of KLRK1 and its relatedness to clinical characteristics, prognosis, and immune infiltration in HNSC using data from The Cancer Genome Atlas (TCGA) database.

## 2. Methods

### 2.1. Data download

We downloaded normal and control samples for head and neck squamous cell carcinoma (HNSCC) from TCGA website at https://portal.gdc.cancer.gov/. A total of 566 samples were downloaded, consisting of 522 tumor samples and 44 normal control samples. Each sample was analyzed for the expression of 59,427 transcripts, including both encoded and non-encoded transcripts. We have downloaded the clinical information of 527 patients with HNSCC from TCGA, among which 520 have gene expression data available. We selected this subset of patients for clinical correlation analysis.

### 2.2. Data extraction and processing procedure

We employed the Perl program to extract the necessary raw data and utilized the R language limma package for differential analysis. To obtain more intuitive statistical results, we incorporated ggplot2 and ggpubr packages to visualize the outcomes. We established 50% as the cutoff point, segmenting samples into high- and low-expression groups. Subsequently, we performed statistical analysis of survival data using the limma and survival packages in the R language. We also designed the Survminer package to create an overall survival (OS) graph, assisting with the visualization of our results. Moreover, the survival ROC package enabled us to conduct ROC analysis and generate corresponding curves. We analyzed the correlation between KLRK1 expression levels and clinicopathological factors using the limma and ggpubr packages in the R language. Simultaneously, we used the ComplexHeatmap package to create a heat map. We performed single- and multiple-factor Cox regression analyses on HNSCC patient prognosis using the survival package. In our study, we utilized TIMER2.0, to explore the relationship between KLRK1 expression levels and immune infiltration, as well as their correlations with other gene expression. CIBERSORT was used to detect the levels of immune cell infiltration in patients with varying pT stages, HPV infections, and primary tumor sites. Subsequently, we conducted data analysis and visualization using R language packages including limma, reshape2, ggpubr, vioplot, and ggExtra.

### 2.3. Statistical analysis

Unless otherwise specified, all significance tests were two-tailed, and a *P* value less than .05 was considered statistically significant.

## 3. Results

### 3.1. Clinicopathologic characteristics of HNSCC selected from TCGA

Table 1 illustrates the clinicopathological characteristics of patients selected from TCGA. We have downloaded the clinical information of 527 patients with HNSCC from TCGA, among which 520 have gene expression data available. We selected this subset of patients for clinical correlation analysis.

**Table 1:** Clinical and pathological characteristics of HNSCC patients selected from TCGA.

Clinicopathological characteristics	Total number: 520	Percentage (%)
Survival status		
Alive	300	57.7
Dead	220	42.3
Age		
>65	178	34.2
<=65	342	65.8
Gender		
Male	384	73.8
Female	136	26.2
Grade		
1	62	11.9
2	304	58.5
3	125	24.0
4	7	1.3
Unknown	22	4.2
AJCC edition		
4th	1	0.2
5th	10	1.9
6th	124	23.8
7th	385	74.0
pStage		
I	27	5.2
II	71	13.7
III	81	15.6
IV	266	51.2
Unknown	75	14.4
pT		
pT0	1	0.2
pT1	48	9.2
pT2	136	26.2
pT3	99	19.0
pT4	174	33.5
Unknown	62	11.9
pN		
pN0	176	33.8
pN1	67	12.9
pN2	169	32.5
pN3	8	1.5
Unknown	100	19.2
cM*		
cM0	489	94.0
cM1	6	1.2
Unknown	25	4.8
HPV status		
Positive	39	7.5
Negative	82	15.8
Unknown	399	76.7
Primary site		
Oral tongue	129	24.8
larynx	117	22.5
Oral cavity	73	14.0
Floor of mouth	62	11.9
Tonsil	43	8.3
Base of tongue	27	5.2
Others	69	13.3
Smoke history		
No	117	22.5
Yes	391	75.2
Unknown	12	2.3
Alcohol history		
No	162	31.2
Yes	347	66.7
Unknown	11	2.1

AJCC = American Joint Committee on Cancer, cM = clinical metastasis, HNSCC = head and neck squamous cell carcinoma, HPV = human papillomavirus, pStage = pathological stage, pT = pathological tumor, pN = pathological node, TCGA = The Cancer Genome Atlas.

* There is only one pathological M1 patient available in this particular dataset. Therefore, we have chosen to analyze the clinical M data for analysis.

### 3.2. KLRK1 is highly expressed in HNSCC and is correlated with the prognosis of HNSCC

**Figure 1.**
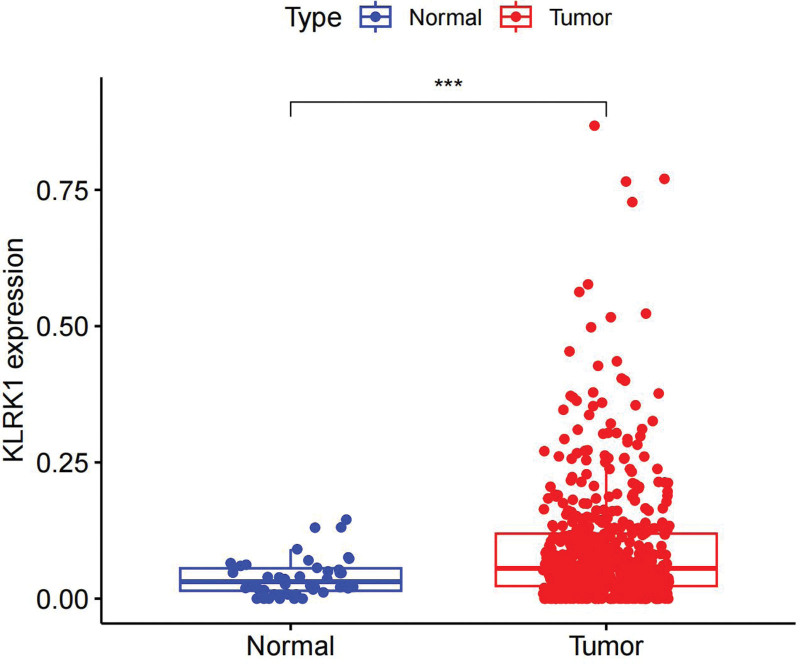
Expression profiles of KLRK1 in HNSCC patients. HNSCC = head and neck squamous cell carcinoma, KLRK1 = killer cell lectin like receptor K1.

Based on the analysis of gene expression differences, it was found that KLRK1 was significantly upregulated in HNSCC tissues compared to normal tissues, and this difference was statistically significant (*P* < .001, Fig. 1).

**Figure 2.**
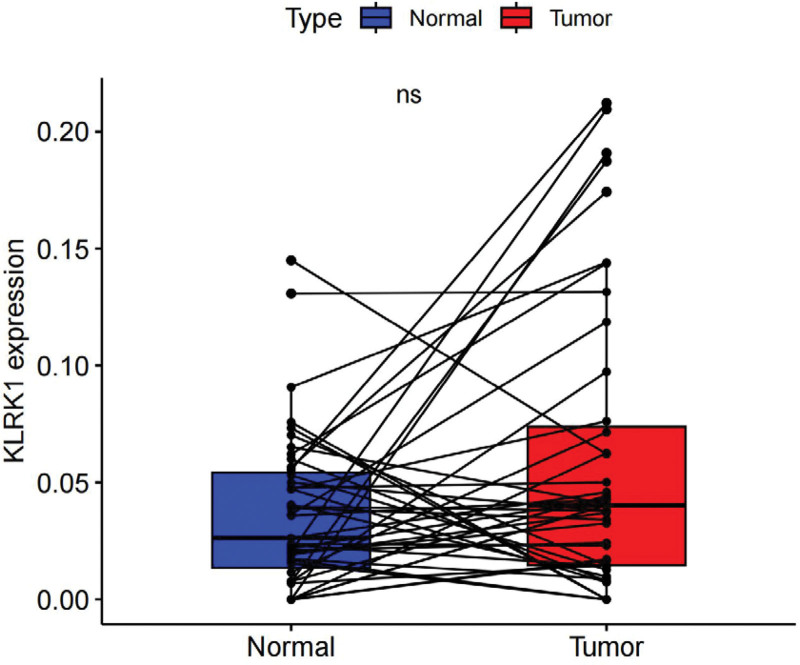
Paired differential expression profiles of KLRK1 in HNSCC patients. HNSCC = head and neck squamous cell carcinoma, KLRK1 = killer cell lectin like receptor K1.

Paired sample differential analysis suggested that there was no significant difference in expression between the normal and cancer tissues (*P* > .05, Fig. 2). However, Figure 2 shows that KLRK1 had a higher average expression level in tumor tissues than in normal tissues. It is possible that the insignificant difference was due to the small number of cases, or that there was no difference.

**Figure 3.**
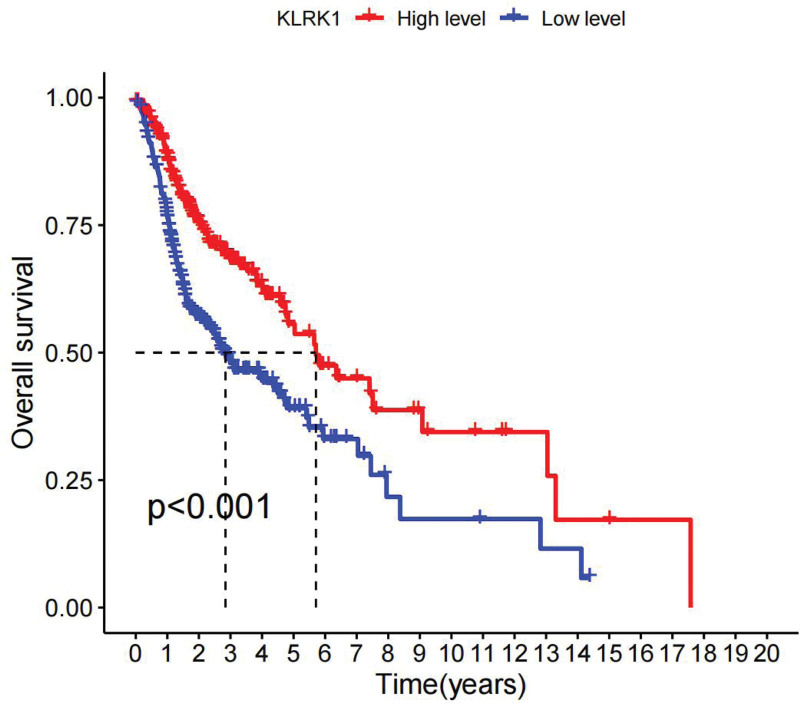
The relationship between the expression of KLRK1 and the prognosis of HNSCC patients. HNSCC = head and neck squamous cell carcinoma, KLRK1 = killer cell lectin like receptor K1.

Regarding survival analysis and prognosis, the results showed that HNSCC patients with low expression levels of KLRK1 had a significantly lower overall survival (OS) rate than those with high expression levels (*P* < .001, as shown in Fig. 3), indicating that low expression of KLRK1 may be a prognostic factor for HNSCC.

**Figure 4.**
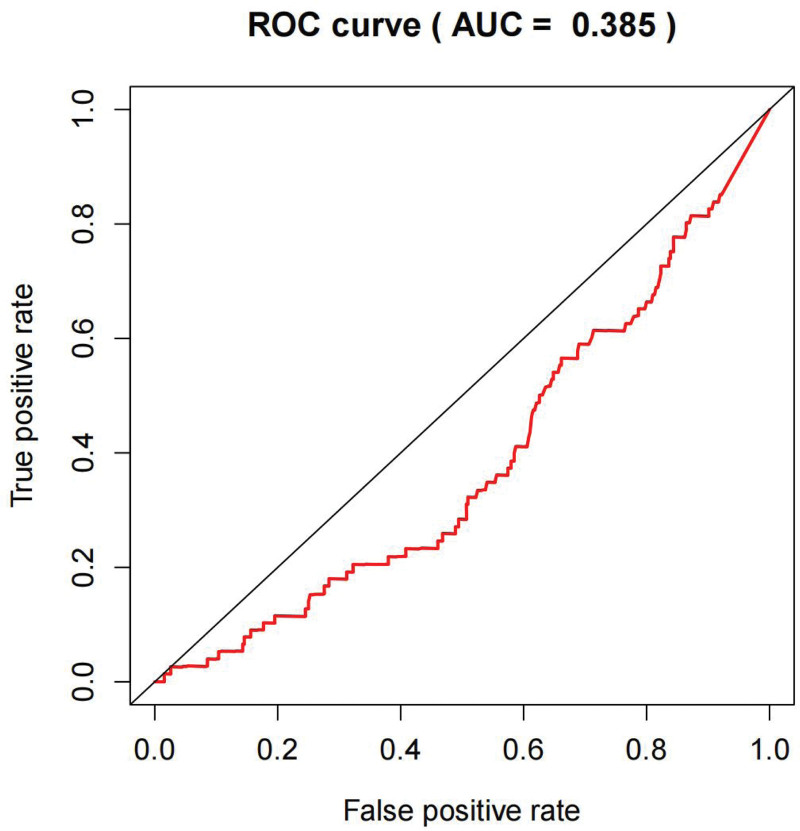
The predictive value of KLRK1 on patient prognosis. All patients were included. AUC = 0.385. KLRK1 = killer cell lectin like receptor K1.

**Figure 5.**
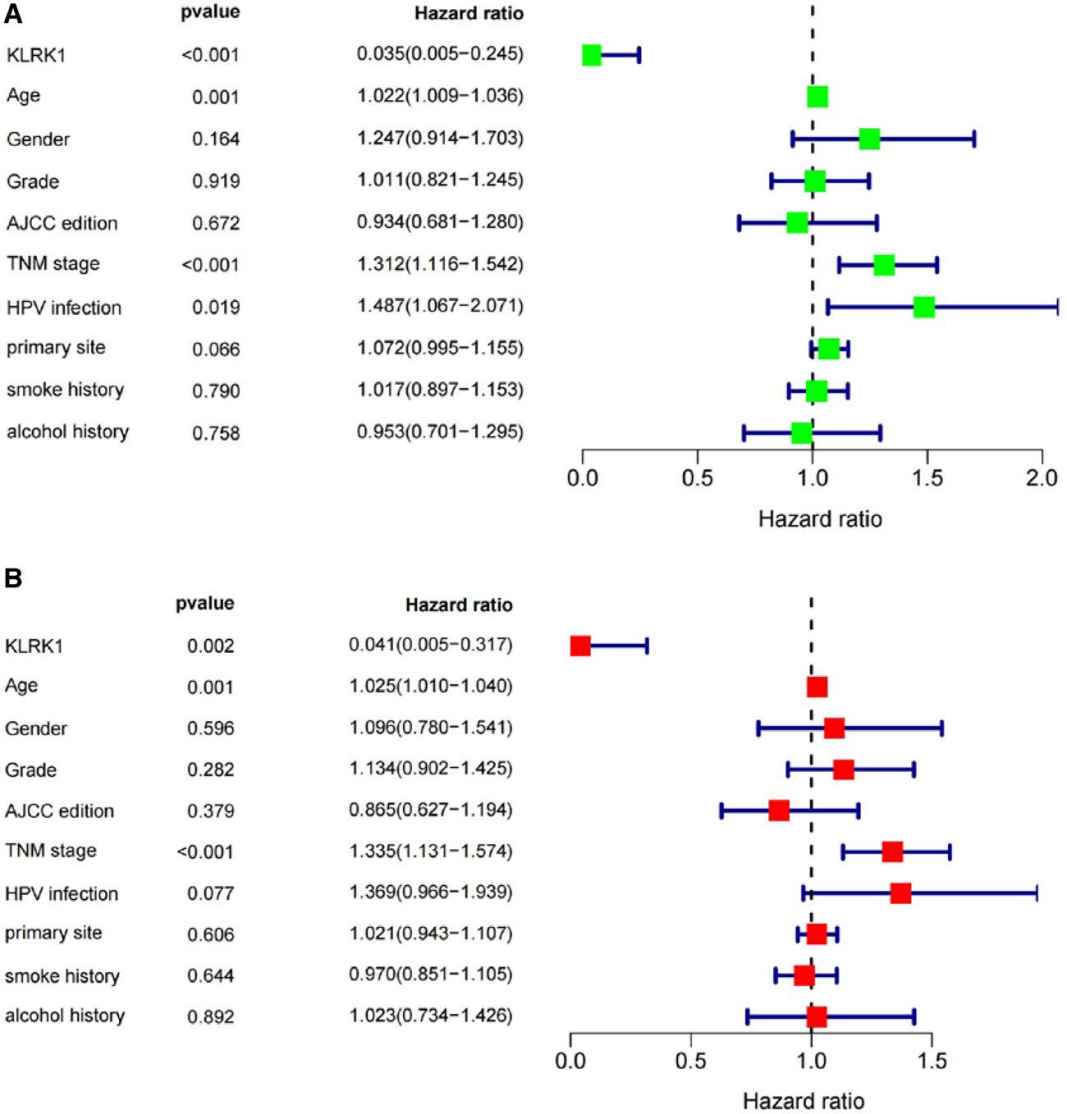
Analyses of KLRK1 and other clinicopathological characteristics. A: Univariate analyses. B: Multivariate analyses. KLRK1 = killer cell lectin like receptor K1.

Moreover, ROC analysis revealed an AUC value of 0.385, suggesting that KLRK1 could be a reliable prognostic indicator (Fig. 4). Further univariate and multivariate analyses indicated that KLRK1 was a key prognostic factor in patients with HNSCC (Fig. 5).

### 3.3. Correlation between KLRK1 gene expression levels and clinicopathological characteristics of HNSCC

**Figure 6.**
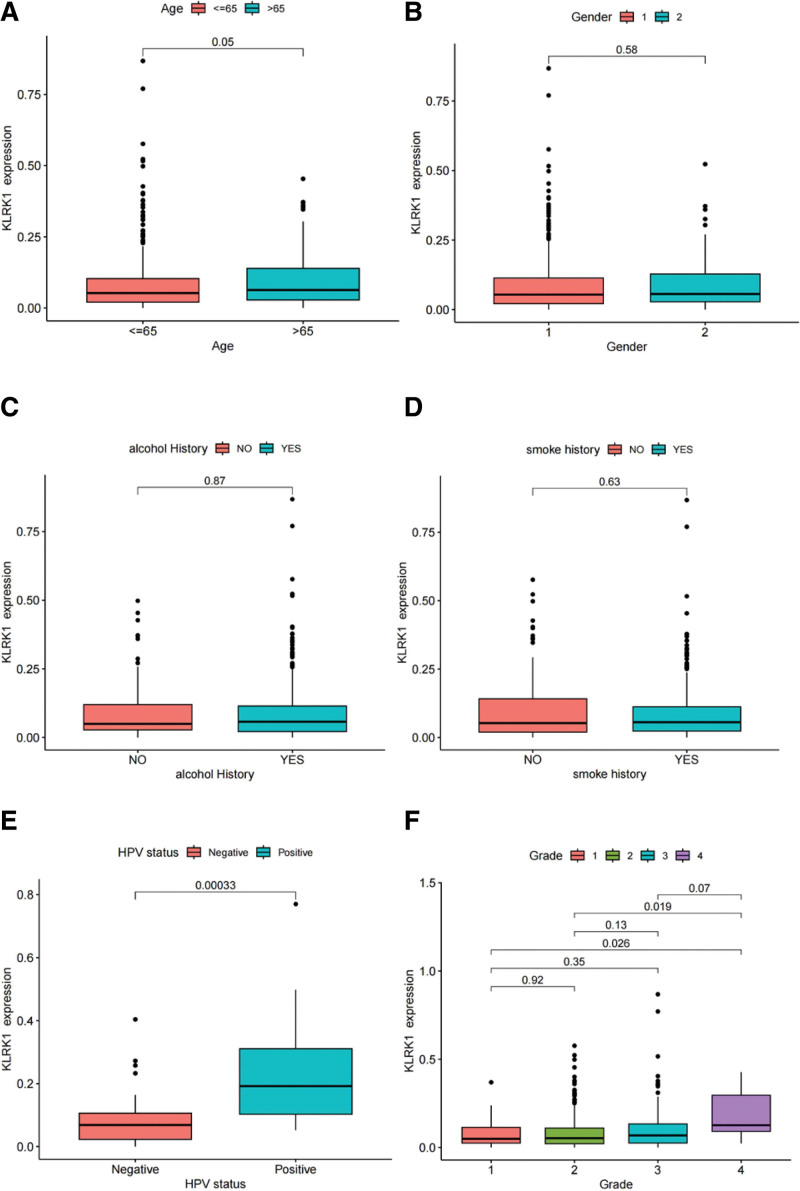
KLRK1 gene expression levels and their correlation with clinicopathological characteristics. A: Age. B: Gender. C: Alcohol History. D: Smoke History. E: HPV Status. F: Histological Grade. G: Survival Status. H: Primary Site. I: pStage. J: pT. K: pN. L: cM. KLRK1 = killer cell lectin like receptor K1.

We analyzed the correlation between KLRK1 gene expression levels and clinicopathological characteristics such as patient survival status, age, sex, histological grading, pathological TNM staging, pT, pN, cM, HPV infection, site of origin, smoking history, and alcohol consumption (Fig. 6A–L). The results showed that KLRK1 expression levels were significantly correlated with patient age (>65 vs <65 years), histological grade (grade 4 vs grade1/2/3), HPV infection, pT (T1 vs T3/4), pN (N0 vs N2/3, N2 vs N3), pTNM (I vs III/IV), site of origin (tonsil vs all other groups, larynx vs oral tongue/oral), and survival status (*P* < .05; Fig. 6A, E–K), but not with sex, cM, smoking history or alcohol consumption (Fig. 6B, C, D, and L). The correlation between KLRK1 and pTNM staging may be related to the pT grading.

### 3.4. KLRK1 gene expression levels and their correlation with immune cells infiltration

**Figure 7.**
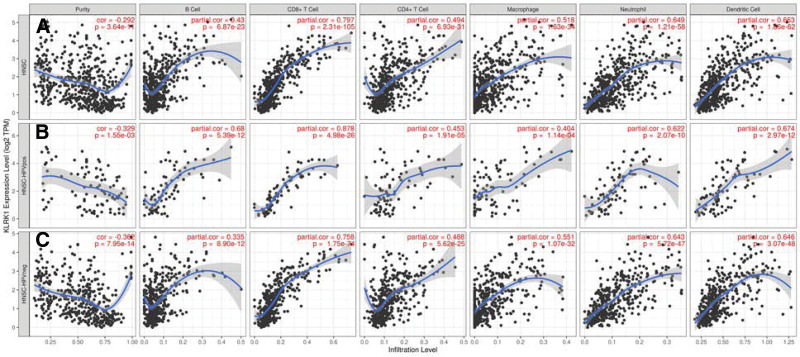
KLRK1 expression levels and their correlation with immune cells infiltration. A: All HNSCC. B: HPV Positive HNSCC. C: HPV Negative HNSCC. HNSCC = head and neck squamous cell carcinoma, KLRK1 = killer cell lectin like receptor K1.

The TIMER2.0 analysis revealed that the expression levels of KLRK1 were significantly associated with the infiltration of various immune cells in HNSCC, particularly CD8 (+) T cells, with a correlation coefficient of 0.797. Additionally, the correlation coefficients between KLRK1 expression levels and CD8 (+) T cell infiltration were 0.878 and 0.758 for HPV (+) and HPV (−) HNSCC, respectively. The correlation coefficients between CD4 (+) T cells and KLRK1 expression were 0.494, 0.453, and 0.488 for HNSCC, HPV (+) HNSCC, and HPV (−) HNSCC, respectively. These results suggest that KLRK1 expression levels are strongly correlated with the level of immune cell infiltration (Fig. 7).

### 3.5. The extent of immune cell infiltration varies across different pT stages, HPV infection statuses, and cases of tonsil cancer

**Figure 8.**
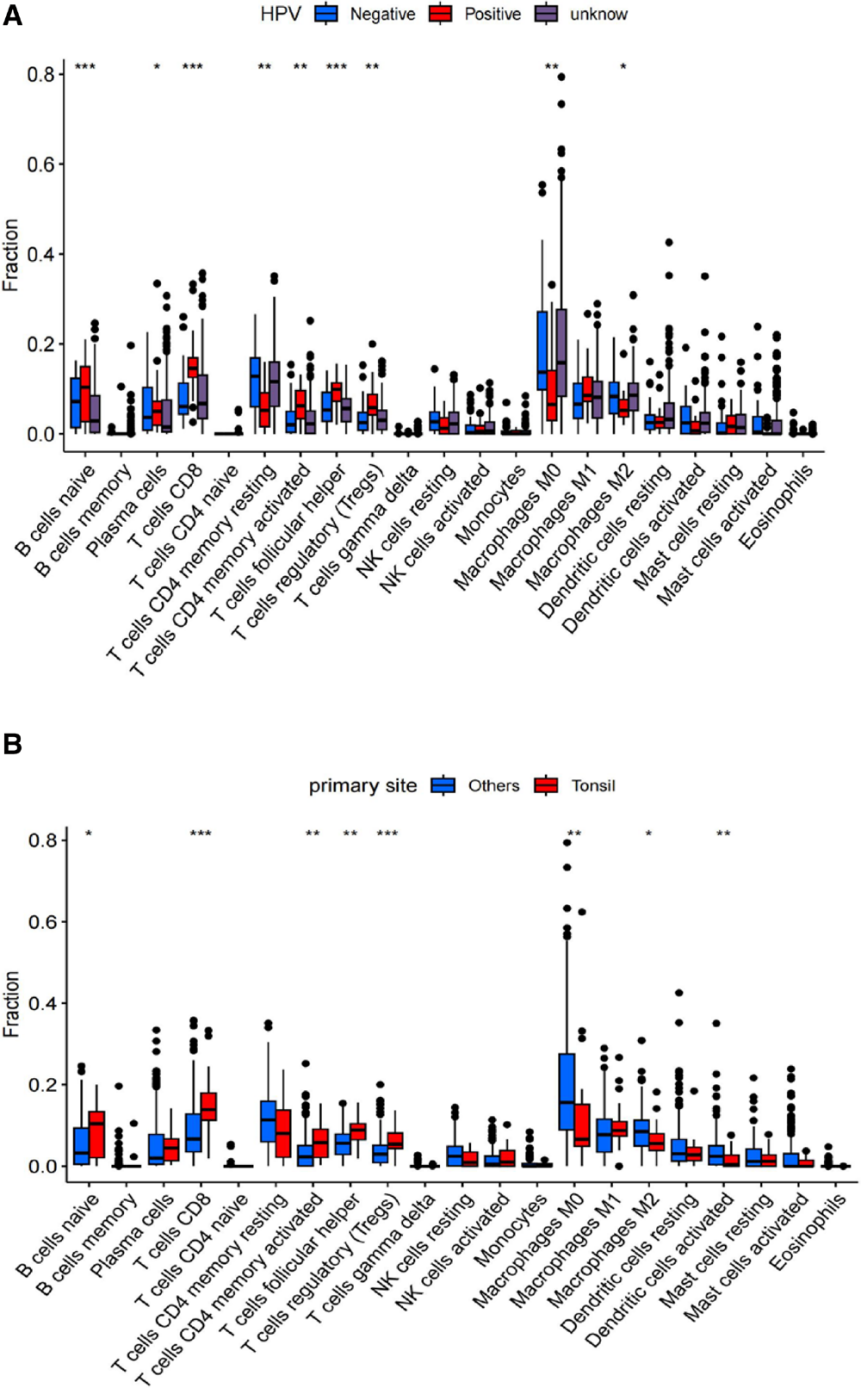
The extent of immune cell infiltration varies across different pT stages, HPV infection statuses, and cases of tonsil cancer. A: HPV infection status. B: primary site. C: pT stages.

KLRK1 expression significantly correlated with pT stage, HPV infection status, and tonsil cancer. The data suggested that immune cell infiltration was higher in cases with lower pT grades, HPV infection, and tonsil cancers, such as CD8 (+) T cells, CD4 (+) memory activated T cells, follicular helper T cells, and regulatory T cells (*P* < .05; subgroups showing *P* < .001, as shown in Fig. 8).

### 3.6. The expression of KLRK1 ligands and their impact on patient prognosis in HNSCC

**Figure 9.**
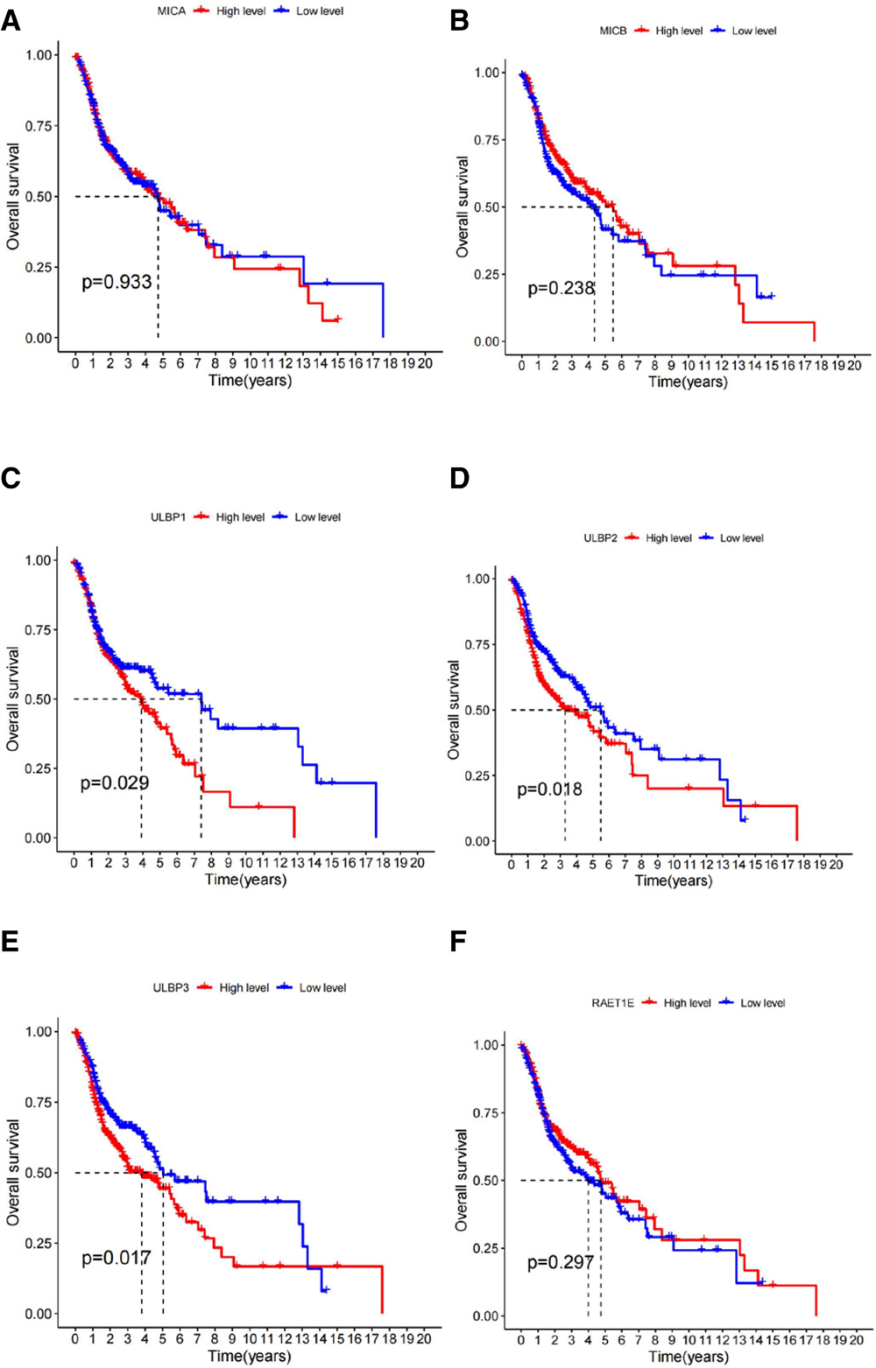
The expression of KLRK1 ligands and their impact on patient prognosis in HNSCC. A: MICA. B: MICB. C: ULBP1. D: ULBP2. E: ULBP3. F: RAET1E. G: RAET1G. H: RAET1L. HNSCC = head and neck squamous cell carcinoma, KLRK1 = killer cell lectin like receptor K1.

**Figure 10.**
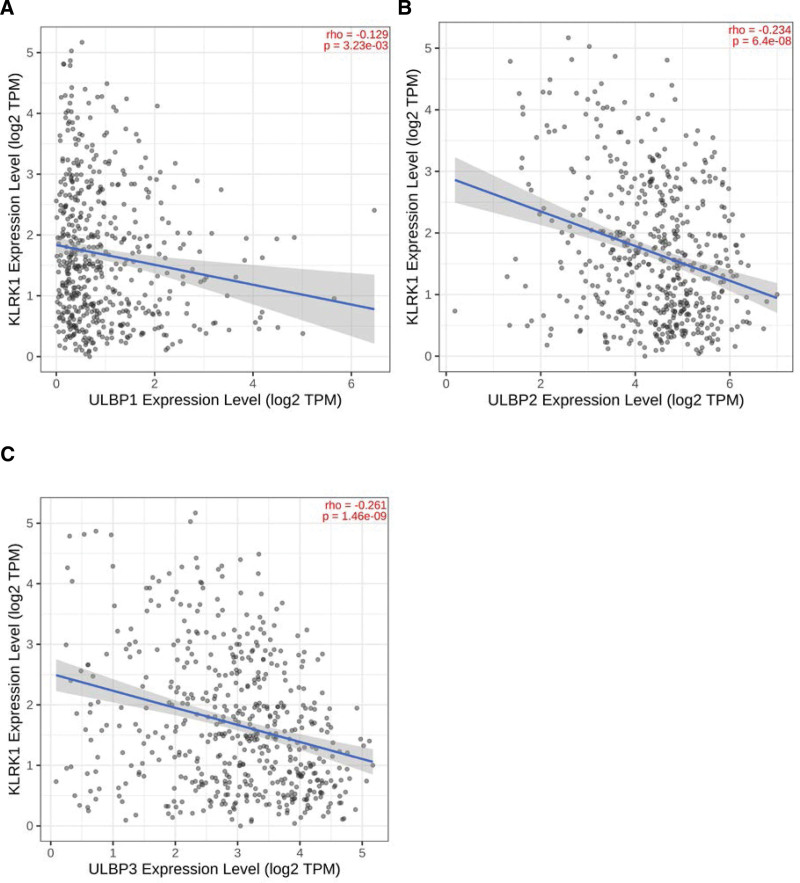
The expression correlation between KLRK1 and ULBP1-3. A: ULBP1. B: ULBP2. C: ULBP3. KLRK1 = killer cell lectin like receptor K1, ULBP = UL16 binding protein.

Our study investigated the expression of KLRK1 ligands and their correlation with prognosis in HNSCC patients. KLRK1 comprises 8 ligands, including MHC class I polypeptide-related sequence A (MICA), MHC class I polypeptide-related sequence B (MICB), and ULBP1-6. In HNSCC, upregulation was observed in MICA, MICB, and ULBP1-4 (*P* < .001), whereas downregulation was observed in ULBP4-5 (*P* < .001 and *P* < .05, respectively). Conversely, no significant difference was observed in ULBP6 expression between tumor and normal tissues. ULBP1-3 are associated with prognosis, and high expression of these ligands increases the risk of death. However, this phenomenon was not observed for other ligands (*P* < .05, Fig. 9). Interestingly, there was a negative correlation between KLRK1 expression and ULBP1-3, highlighted by correlation coefficients of −0.129, −0.234, and −0.261, respectively (*P* < .001, Fig. 10).

### 3.7. The prognostic relationship between expression level of KLRK1 and its ligands ULBP1-3 in patients with tonsil cancer

**Figure 11.**
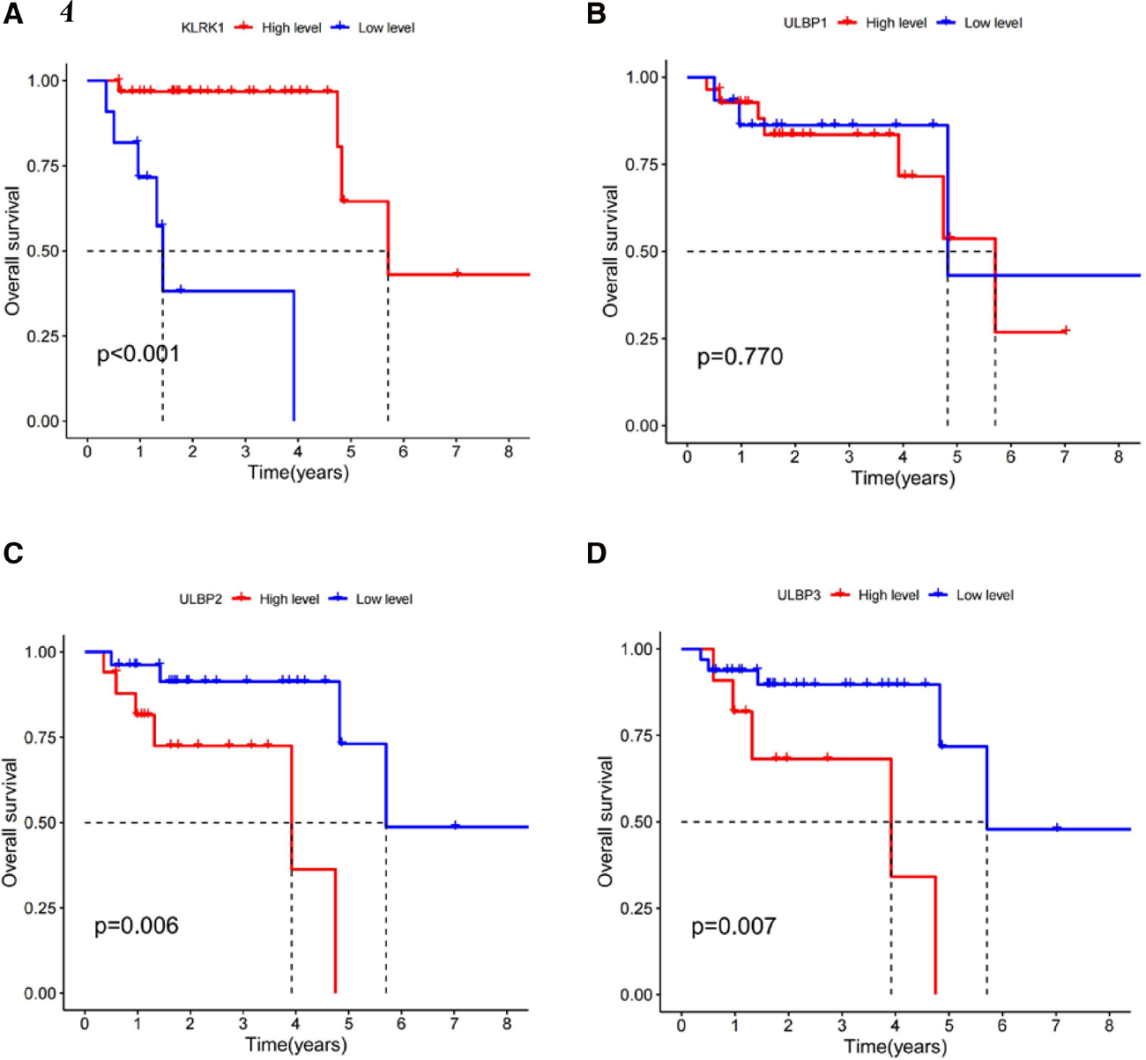
Prognostic relationship between expression level of KLRK1 and ULBP1-3 in tonsil cancer. A: KLRK1. B: ULBP1. C: ULBP2. D: ULBP3. KLRK1 = killer cell lectin like receptor K1, ULBP = UL16 binding protein.

The expression level of KLRK1 in tonsil cancers was significantly higher than in other head and neck squamous cell carcinomas (Fig. 6H). In this context, we analyzed the prognostic relationship between the expression levels of KLRK1 and ULBP1-3 in tonsil cancers. Patients with low expression of KLRK1 or high expression of ULBP2/3 had worse prognosis than those with low expression of ULBP2/3 (*P* < .01, Fig. 11A, C, and D), whereas the expression level of ULBP1 had no significant effect on the prognosis of tonsil cancer (*P* = .770, Fig. 11B). This finding suggests the potential prognostic value of KLRK1 and ULBP2/3 expression levels in tonsil cancer and highlights the importance of further investigation of the role of KLRK1 and its ligands in the development and progression of this type of cancer.

### 3.8. The prognostic relationship between expression level of KLRK1 and its ligands ULBP1-3 in patients with laryngeal cancer

**Figure 12.**
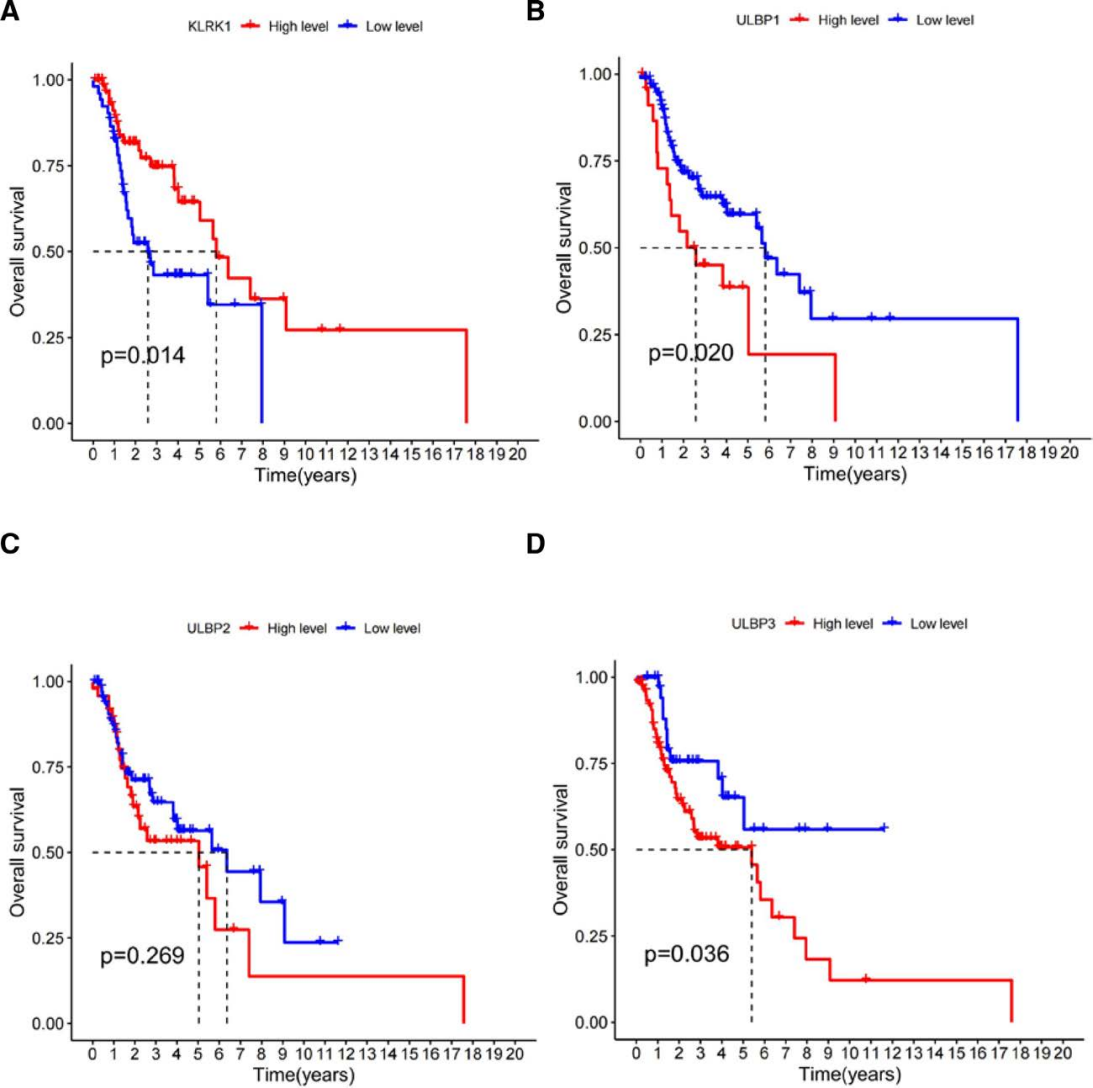
Prognostic relationship between expression level of KLRK1 and ULBP1-3 in laryngeal cancer. A: KLRK1. B: ULBP1. C: ULBP2. D: ULBP3. KLRK1 = killer cell lectin like receptor K1, ULBP = UL16 binding protein.

The expression level of KLRK1 was lower in laryngeal cancer than that in tonsil cancer (Fig. 6H). Therefore, we conducted an analysis to examine the relationship between the expression levels of KLRK1 and its ligands ULBP1-3 in patients with laryngeal cancer. Our findings revealed that patients with high expression levels of KLRK1 had a better prognosis than those with low expression levels of KLRK1 (*P* < .05, Fig. 12A). In contrast, patients with high expression levels of ULBP1/3 had a worse prognosis than those with low expression levels of ULBP1/3 (*P* < .05, Fig. 12B and D). Notably, we observed no significant correlation between the expression level of ULBP2 and prognosis (*P* = .269, Fig. 12C), suggesting that ULBP2 may not be a significant prognostic factor for laryngeal cancer.

## 4. Discussion

In our study, we found the following expression characteristics of KLRK1: the expression levels of KLRK1 in tumor tissues were not consistently higher than those in normal tissues. Although the average level of KLRK1 expression in tumor tissues was higher than that in normal tissues, paired analysis of 44 samples showed no significant difference in expression. This could have been due to negligible differences in expression levels or the small sample sizes. KLRK1 expression increased in HPV-positive patients. Smaller tumor tissues tend to have higher expression levels of KLRK1. The expression levels of KLRK1 vary greatly between different types of cancer, with higher levels observed in tonsil cancer than in other types of cancer. Immune cell infiltration was also significantly higher in tonsil cancer, which may be related to tonsil being an immune organ. Combined with the overall better prognosis of tonsil cancer compared to other cancers, this suggests that a higher degree of immune infiltration could influence patient prognosis.

After analyzing the data, it was discovered that the high expression of KLRK1 in HNSCC was mainly due to CD4/8 (+) T lymphocytes rather than NK cells. This finding suggests that targeted immunotherapy to enhance NK cell killing may not achieve optimal efficacy. Instead, treatment plans that increase the activity of CD4/8 (+) T lymphocytes could be more effective. In a clinical trial for HNSCC, patients treated with 1,25(OH)(2)D(3) had increased levels of CD4/8 (+)T lymphocytes in HNSCC tissue, which was associated with a better prognosis.^[14]^ Therefore, increasing the vitality or infiltration level of CD4/8 (+)T lymphocytes is recommended to improve patient prognosis.

The expression level of KLRK1 was significantly different between the HPV-positive and HPV-negative patients. One possible reason for this is that HPV-positive cancer patients are primarily concentrated in the tonsil, which is an immunological organ. The degree of immune cell infiltration in tonsil cancer is typically higher than that observed in other types of cancers.^[15]^ Our study revealed that tonsil cancers exhibit a higher degree of immune cell infiltration than other squamous cell carcinomas in the head and neck region. Interestingly, this difference was particularly noticeable for immune cells such as CD8 (+) T cells, CD4 (+) memory activated T cells, follicular helper T cells, and regulatory T cells. Further analysis of patients who were not pT-staged suggested that there were significant variations in KLRK1 expression and immune cell infiltration levels. Strikingly, our findings showed that the lower the tumor T grade, the higher the expression level of KLRK1 and the intensity of immune cell infiltration. Together, these outcomes support the notion that the level of immune cell infiltration could be a crucial determinant of patient prognosis.

Our study revealed that the impact of KLRK1 extended beyond patient prognosis and immune cell infiltration but also to its ligand expression, particularly in patients with prognostic value. The interaction between KLRK1 and its ligands in the tumor microenvironment is complex. Tumor cells had the ability to secrete soluble NKG2DLs, which compete with KLRK1 on the immune cell surface. This competition allows tumor cells to escape immune surveillance.^[16]^ Lee et al^[17]^ concluded that malignant gliomas were able to escape KLRK1-mediated immune surveillance mainly by suppressing the expression of NKG2DLs, such as MICA and ULBP2, via an autocrine TGF-β loop and MP-dependent cell surface shedding. Compared to other NKG2DLs, their discovery suggests that the loss of MICA and ULBP2 plays a more significant role in glioma immune escape.

**Figure 13.**
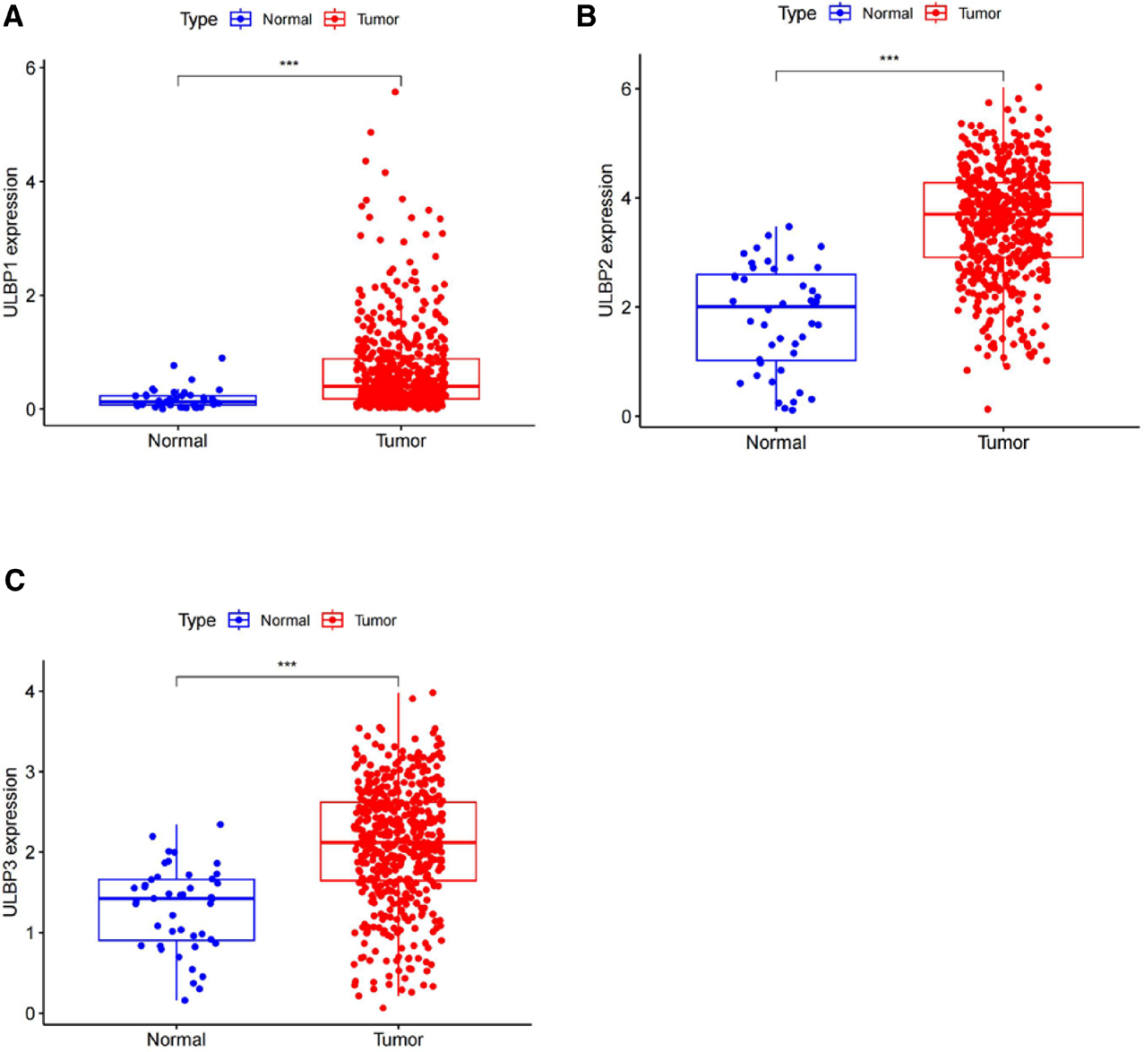
Expression of ULBP1-3 in tumors and normal tissues of HNSCC. A: ULBP1. B: ULBP2. C: ULBP3. HNSCC = head and neck squamous cell carcinoma, ULBP = UL16 binding protein.

Our study revealed that ULBP1-3 is a prognostic marker for HNSCC. High expression of these ligands in HNSCC increased the risk of patient death, especially in tonsil and larynx cancers (Figs. 11 and 12), and their expression was negatively correlated with KLRK1 expression (Fig. 10). In tonsil and laryngeal cancers, the immune escape mechanism mediated by KLRK1 and its ligands ULBP1-3 may competitively inhibit KLRK1, owing to insufficient expression/immune cell infiltration of KLRK1 or sufficient secretion of soluble NKG2DLs by tumor cells. These mechanisms differ among the HNSCC locations. In HNSCC, including tonsil and laryngeal cancers, ULBP2 had the highest expression level, ULBP1 had the highest, lowest, and ULBP3 was in between (Fig. 13). In tonsil cancer, tumor cells already showed high expression of KLRK1 (Fig. 6H), and patients with high expression of KLRK1 had a significantly better prognosis than those with low expression of KLRK1 (Fig. 11A). At the same time, we found that patients with high expression of ULBP2/3 in tonisil cancer had a poor prognosis (Fig. 11C and D), whereas high expression of ULBP1 was unrelated to prognosis (Fig. 11B). Therefore, we speculate that the possible immune evasion mechanism in tonsil cancer is that tumor cells produce more soluble NKG2DLs (such as ULBP2/3) to competitively inhibit the high expression of KLRK1, and inadequate expression of ULBP1 cannot produce sufficient soluble ULBP1 for immune evasion (the expression level of ULBP1 is much lower than that of ULBP2/3) (see Fig. 13). In laryngeal cancer, the expression level of KLRK1 was lower, and patients with low KLRK1 expression had a significantly worse prognosis than those with high KLRK1 expression (Fig. 12A). Meanwhile, we found that low expression of ULBP1/3 was associated with good prognosis (Fig. 12B and D), whereas the difference in ULBP2 expression was unrelated to prognosis (Fig. 12C). Therefore, we speculate that a possible immune evasion mechanism in laryngeal cancer is that tumor cells inhibit the low expression of KLRK1 by producing soluble NKG2DLs, and that a lower level of soluble NKG2DLs can lead to immune evasion. The overall expression level of ULBP2 in tumor cells is already high (the expression level of ULBP2 is much higher than that of ULBP1/3, see Fig. 13), and tumor cells with low expression of ULBP2 can produce sufficient soluble ULBP2 for immune evasion; therefore, there is no difference in prognosis between patients with high expression of ULBP2 and those with low expression of ULBP2. These hypotheses require further follow-up experiments to verify whether more targeted and effective drugs can be developed, consistent with our speculation. For HNSCC patients with different expression levels of KLRK1 and its ligands, selective development of more effective targeted drugs may include targeting ULBP2/3 rather than upregulating KLRK1 or inhibiting ULBP1 for tonsil cancer and targeting ULBP1/3 rather than inhibiting ULBP2 for laryngeal cancer. This discovery presents a novel therapeutic target for developing drugs that can mitigate the ability of tumor cells to evade immune surveillance by targeting soluble KLRK1 ligands. These drugs may offer promising new avenues for treating cancer and for improving patient outcomes.

However, our study may have the following limitations. Firstly, this study only investigated the role of KLRK1 and ULBP1-3 inHNSCC, and other types of cancers may have different immune escape mechanisms. Therefore, our conclusions may not be generalizable to other types of cancers. Secondly, our study is based on retrospective analysis, which may introduce certain observational biases. Further prospective follow-up studies can provide more accurate and reliable results. In such studies, more rigorous research designs and methods can be employed to optimize the reliability and validity of the results. Furthermore, we have made preliminary speculations about the interaction mechanisms between KLRK1 and ULBP1-3, but further experimental verification is still needed. Through in-depth experimental research, the detailed interactions between these molecules can be explored, and their specific roles in tumor immune escape processes can be understood. Finally, our study sample is relatively limited, and it is necessary to validate the results in a larger population to ensure their reliability. Increasing the sample size can improve the statistical power of the study and reduce result errors due to sample biases. In future research, recruiting more participants can strengthen the support and stability of the research findings.

## 5. Conclusion

Head and neck squamous cell carcinoma (HNSCC) is a common malignant tumor that frequently occurs in the head and neck region, and has a low 5-year survival rate. Despite significant advances in immunotherapy, its efficacy in HNSCC treatment remains unsatisfactory. Killer cell lectin-like receptor K1 (KLRK1), a marker highly expressed in immune cells, can bind to ligands expressed on cancer cells to exert its antitumor effect. However, the role of KLRK1 in HNSCC has not been extensively studied. This study aimed to investigate the role of KLRK1 in immune infiltration and its correlation with prognosis in HNSCC, analyze KLRK1 expression data obtained from the TCGA database, and study the relationship between KLRK1 and immune infiltration. Furthermore, the prognostic correlation between the expression levels of KLRK1 and its ligands in HNSCC patients was analyzed. The results revealed that KLRK1 was highly expressed in HNSCC and correlated with better prognosis. The expression of KLRK1 was associated with various factors, including age, histological grade, HPV infection, pT, pN, pTNM stage, primary site, and survival status. KLRK1 overexpression is closely associated with high levels of immune cell infiltration, particularly in CD4/8 (+) T lymphocytes. Among the KLRK1 ligands, the expression of UL16-binding proteins (ULBP) 1-3 was higher and was associated with an increased risk of death. Furthermore, KLRK1 expression was negatively correlated with the expression of ULBP1-3. In patients with tonsil cancer, those with a high expression level of ULBP2/3 had a worse prognosis, whereas the opposite was true for those with a low expression level, possibly because of the expression of more soluble ligands. In laryngeal cancer, the expression levels of ULBP1/3 correlated with poor prognosis (*P* < .05), whereas ULBP2 expression was not significantly correlated with prognosis (*P* = .269). Hence, this study revealed that KLRK1 is highly expressed in HNSCC and is associated with a better prognosis and immune infiltration. Patients overexpressing KLRK1 ligands had a worse prognosis, which may be due to the expression of more soluble ligands.

## Author contributions

**Conceptualization:** Jiaxin Qian, Shaoyan Liu, Liyuan Wei, Wensheng Liu.

**Formal analysis:** Haosheng Tan.

**Funding acquisition:** Wensheng Liu.

**Investigation:** Wensheng Liu.

**Methodology:** Dangui Yan, Liyuan Wei.

**Project administration:** Haosheng Tan, Jiaxin Qian, Wensheng Liu.

**Software:** Haosheng Tan, Huaiyu Yang.

**Supervision:** Liyuan Wei, Wensheng Liu.

**Validation:** Huaiyu Yang, Shaoyan Liu.

**Visualization:** Huaiyu Yang.

**Writing – original draft:** Haosheng Tan.

**Writing – review & editing:** Dangui Yan, Wensheng Liu.
